# Creation of a Latin-American Dementia Advance Care Planning Guide

**DOI:** 10.1093/geroni/igab046.3583

**Published:** 2021-12-17

**Authors:** Moroni Fernandez Cajavilca, Lauren Solkowski, Rebecca Utz, Ana Sanchez-Birkhead, Nancy Aruscavage, Katherine Supiano, Eli Iacob, Kara Dassel

**Affiliations:** 1 University of Utah Hospital, West Valley City, Utah, United States; 2 University of Utah, University of Utah, Utah, United States; 3 University of Utah, Salt Lake City, Utah, United States

## Abstract

The LEAD Guide (Life-Planning in Early Alzheimer’s and Dementia) is an advance care planning conversation guide for use within the context of dementia (Dassel et al., 2019). Considering that Latino adults have the highest risk of ADRD, a culturally sensitive and translated Spanish version of the LEAD Guide was needed. Therefore, the objective of this study was to: 1) translate the LEAD Guide into a Latin-American Spanish version (i.e., LA LEAD) and 2) assess the applicability and acceptability of the LA LEAD through focus groups with Latino older adults. First, the LEAD Guide was translated into a “neutral” Spanish version. Second, forward and backward translation was conducted to create the LA LEAD. Third, two 1.5-hour focus groups with Spanish-speaking Latino adults age 50+ who were: a) healthy adults (N=7) or b) current or previous dementia caregivers (N=7) were held. The focus groups were recorded, translated, and transcribed for descriptive analysis, which revealed three domains regarding the LA LEAD: 1) Family Dynamics: the guide could help prevent family conflict, designate a health care proxy, and reduce burden; 2) Cultural Expectations: acknowledgement of cultural nuances between LA countries, the familial responsibility of caring for family in the home, and the influence of religion on end-of-life care decisions, and 3) Health Literacy: lack of knowledge about advance care planning conversations, documentation, and dissemination. This process resulted in the creation of a validated LA LEAD Guide, which is a culturally and linguistically appropriate and acceptable advance care planning tool for Latino older adults.

